# HIV-associated penile anaerobes disrupt epithelial barrier integrity

**DOI:** 10.1371/journal.ppat.1013094

**Published:** 2025-04-17

**Authors:** Lane B. Buchanan, Zhongtian Shao, Ronald M. Galiwango, Shirley Constable, David Zuanazzi, Victoria Menya Biribawa, Henry Rogers Ssemunywa, Annemarie Namuniina, Brenda Okech, Gabriella Edfeldt, Annelie Tjernlund, Aaron A. R. Tobian, Daniel E. Park, Tony Pham, Maliha Aziz, Juan E. Salazar, Sydney Nelson, Cindy M. Liu, Rupert Kaul, Jessica L. Prodger

**Affiliations:** 1 Department of Microbiology and Immunology, Schulich School of Medicine and Dentistry, Western University, London, Ontario, Canada; 2 Rakai Health Sciences Program, Rakai, Uganda; 3 Department of Medicine, University Health Network, Toronto, Ontario, Canada; 4 Uganda Virus Research Institute, International AIDS Vaccine Initiative, Entebbe, Uganda; 5 Department of Medicine Solna, Karolinska Institutet, Stockholm, Sweden; 6 Department of Microbiology, Tumor and Cell Biology, Karolinska Institutet, Stockholm, Sweden; 7 Department of Pathology, Johns Hopkins University School of Medicine, Johns Hopkins University, Baltimore, Maryland, United States of America; 8 Department of Environmental and Occupational Health, Milken Institute School of Public Health, George Washington University, Washington District of Columbia, United States of America; 9 Department of Epidemiology and Biostatistics, Schulich School of Medicine and Dentistry, Western University, London, Ontario, Canada; NIH, NIAID, UNITED STATES OF AMERICA

## Abstract

Specific anaerobic taxa within the penile microbiome—the Bacteria Associated with Seroconversion, Inflammation and Immune Cells (BASIC) species—enhance HIV-1 susceptibility, in part by recruiting susceptible cells to the inner foreskin. However, their effect on epithelial barrier integrity has not been described. Using foreskin tissues and penile swabs from 116 males undergoing voluntary medical male circumcision, we assessed the relationship between BASIC species and foreskin epithelial thickness, junction protein expression, and cellular proliferation. The absolute abundance of BASIC species was associated with reduced tissue expression of the epithelial junction proteins claudin-1 and E-cadherin, and with elevated soluble E-cadherin in penile secretions, suggesting proteolytic cleavage. These effects were not seen in participants with a high abundance of control taxa without high levels of BASIC species. The BASIC species *Prevotella bivia*, but not *Peptostreptococcus anaerobius* or *Dialister micraerophilus*, was shown to directly degrade recombinant human E-cadherin and to increase the release of soluble E-cadherin from foreskin epithelial cells *in vitro*. *In vivo* BASIC species absolute abundance was also linked to a thicker nucleated epithelium and increased keratinocyte proliferation, with no change in *stratum corneum* thickness. Therefore, BASIC species may enhance penile HIV susceptibility by directly disrupting epithelial integrity, in addition to previously described target cell recruitment.

## Summary

Specific anaerobic bacteria in the penile microbiome are associated with increased risk of HIV acquisition. In a study of 116 Ugandan males, we now link these bacteria to epithelial barrier disruptions. In participants with a high abundance of these anaerobic bacteria, we found reduced expression of epithelial barrier proteins E-cadherin and claudin-1, and elevated soluble E-cadherin, a biomarker of epithelial barrier disruption. We found that one species, *Prevotella bivia*, and its conditioned media, could directly degrade E-cadherin and increase soluble E-cadherin release from primary epithelial cells *in vitro*. These same anaerobic bacteria were also linked to epithelial thickening and increased keratinocyte proliferation, effects commonly seen in inflammatory skin conditions. Taken together, our results provide evidence that *Prevotella bivia* can directly cleave epithelial proteins and cause epithelial changes that may facilitate HIV entry into penile tissue. Targeting these bacteria therefore holds promise as a preventative therapy for HIV, in addition to other sexually transmitted infections.

## Introduction

Voluntary medical male circumcision (MMC) reduces the risk of human immunodeficiency virus 1 (HIV) acquisition through insertive vaginal sex by at least 60% [1–3]. This is due, at least in part, to the reduction of specific taxa of anerobic bacteria from the penis, including *Peptostreptococcus anaerobius*, *Prevotella bivia*, *Prevotella disiens*, *Dialister propionicifaciens*, *Dialister micraerophilus*, and a genetic near neighbor of *Dialister succinatiphilus*. These bacteria are also known as Bacteria Associated with HIV Seroconversion, Inflammation, and Immune Cells, or BASIC species [4]. Current evidence suggests that BASIC species increase HIV susceptibility through the induction of local inflammation, resulting in the recruitment of immune cells bearing the HIV receptors CD4 and CCR5 to the foreskin where they may be exposed to HIV in genital secretions during sex [4]. The density of cells expressing these receptors at the site of viral exposure is a key determinant of simian immunodeficiency virus (SIV) transmission in macaques [5]. These data suggest that immune cell recruitment from BASIC species may increase HIV transmission.

However, to access these susceptible immune cells HIV must traverse the stratified squamous epithelium of penile skin. First, virions must cross the *stratum corneum*, the outermost layer of the epithelium comprised of anuclear flattened corneocytes. These cells, connected by corneodesmosomes, contain filamentous keratin and are surrounded by a hydrophobic lipid matrix [6]. The remainder of the epithelium is comprised of nucleated keratinocytes which originate from proliferating cells in the *stratum basale* and form the *stratum spinosum* and *stratum granulosum* layers as they differentiate. Nucleated keratinocytes in these layers are held tightly together by cell-cell junctions including tight junctions, adherens junctions, and desmosomes.

Tight junctions provide a physical barrier between epithelial cells, limiting the passage of ions, solutes, and pathogens [7]. Claudin proteins form the backbone of tight junctions [8], with claudin-1 in particular being essential for skin barrier function [9]. Adherens junctions provide cohesion between epithelial cells through homophilic binding of cadherin proteins. In the skin, this cadherin is E-cadherin, which plays the important role of determining tissue architecture and morphology by regulating the actin cytoskeleton [7]. Desmosomes, like adherens junctions, provide cohesion between epithelial cells, but also contribute robust mechanical strength to epithelial tissue [10]. One critical desmosome protein in the skin is desmoglein-1; mice deficient in this protein die from skin blistering and loss of epithelial barrier function [11], and autoantibodies against desmoglein proteins in humans can cause pemphigus, a severe blistering disease [12]. Together, the different cell-cell junctions provide physical strength to the skin, impede pathogen entry, and regulate solute diffusion.

Epithelial junction integrity can be disrupted by invading pathogens or by immune responses. Bacterial virulence factors including pore-forming toxins, cytoskeleton-modifying proteins, and lipopolysaccharide can all disrupt the epithelial barrier [13], as can the proteases and reactive oxygen species that are released from immune cells in response to pathogens. Since epithelial junction proteins are a key component of epithelial barrier function, their disruption may facilitate HIV penetration into foreskin tissue, enabling access to susceptible immune cells in the epidermis and underlying dermis.

We hypothesized that BASIC species alter epithelial barrier integrity in the foreskin, which may in turn facilitate HIV tissue penetration and contribute to increased HIV acquisition in uncircumcised men [4]. In this study, we explored associations between the absolute abundance of BASIC species and characteristics of the foreskin epithelium.

## Results

### Participant demographics

Detailed participant demographics have been previously described [14]. Briefly, the median age of participants was 24 years (range 18–49 years), and just over half (60; 52%) were married or cohabiting with a female partner. All participants reported having previously had insertive vaginal sex, with 44 (38%) having reported partaking in vaginal sex in the week prior to enrolment. Most participants (102; 88%) reported retracting the foreskin to wash the penis at least daily. Of the 116 participants, 25 were randomized to the no treatment arm and did not receive any antibiotic (22%), while the remaining 91 men were randomized to a treatment course of either topical metronidazole (23; 20%), topical clindamycin (22; 19%), topical hydrogen peroxide (23; 20%), or oral tinidazole (23; 20%) before MMC. Participant demographics and antimicrobial treatments by bacterial grouping are described in **Table 1**. The effect of antimicrobial treatments on BASIC species absolute abundance and foreskin inflammation have been previously reported [14]. Briefly, topical antimicrobial treatments, including clindamycin and metronidazole, reduced the absolute abundance of BASIC species but increased control taxa at the time of MMC. Topical metronidazole was also associated with increased tissue E-cadherin and decreased soluble E-cadherin. Reductions in BASIC species correlated with reductions in soluble E-cadherin at all time points [14].

**Table 1:** Participant demographics by bacterial grouping.

	No BASIC^†^ (n = 25)	High Control (n = 21)	High BASIC^†^ (n = 21)	Overall (n = 116)
Age, years (mean; range)	25 (18-36)	26 (18-40)	26 (19-49)	26 (18-49)
Married or cohabiting (n, %)	11 (44%)	11 (52%)	15 (71%)	60 (52%)
>1 sexual partner in past 6 months (n, %)	10 (40%)	5 (24%)	11 (52%)	53 (46%)
Vaginal sex in past week (n, %)	7 (28%)	5 (24%)	11(52%)	44 (38%)
Wash penis at least daily (n, %)	19 (76%)	20 (95%)	20 (95%)	102 (88%)
Treatment Group (n, %)				
No Treatment	2 (8%)	2 (10%)	10 (48%)	25 (22%)
Oral tinidazole	2 (8%)	5 (24%)	3 (14%)	23 (20%)
Topical metronidazole	8 (32%)	4 (19%)	3 (14%)	23 (20%)
Topical clindamycin	12 (48%)	4 (19%)	0 (0%)	22 (19%)
Topical hydrogen peroxide	5 (8%)	2 (10%)	5 (24%)	23 (20%)

† BASIC: Bacteria Associated with Seroconversion, Inflammation, and immune Cells

### BASIC species are associated with altered expression of epithelial junction proteins

**Fig 1:**
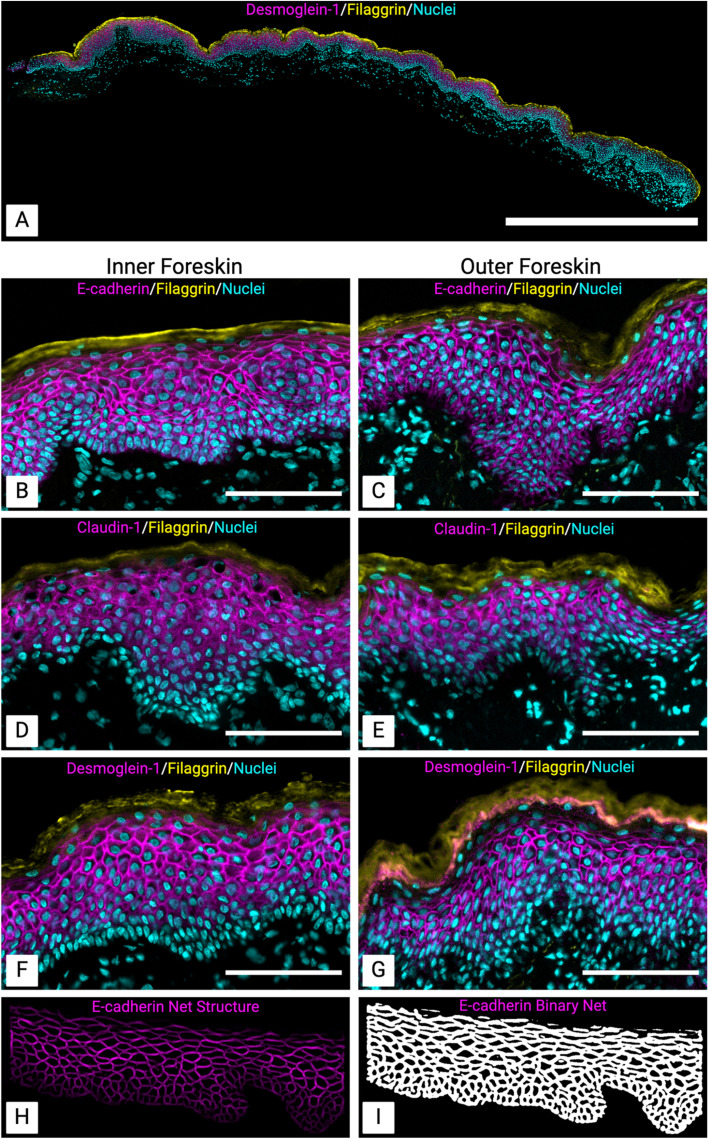
Representative immunofluorescence images of staining and quantification of epithelial junction proteins. Inner and outer foreskin tissue, showing a representative full tissue section (A), and staining (magenta) for junction proteins E-cadherin (B, C), claudin-1 (D, E), and desmoglein-1 (F, G). The *stratum corneum* was visualized by staining for filaggrin (yellow) and cell nuclei are shown in cyan (DAPI). A neuriteness filter was applied to create the net structure (H), and a threshold was applied to generate a binary net (**I**) for quantification. Image scale bars are 1000 µm (A) and 100 µm (B–G). Brightness and contrast have been enhanced from the original images for visualization purposes.

Immunofluorescence was performed on inner and outer foreskin tissues from each participant. Images of full tissue sections (**Fig 1A**) were used to quantify the relative fluorescence of epithelial junction proteins E-cadherin (**Fig 1B** **and 1C**), claudin-1 (**Fig 1D and 1E**), and desmoglein-1 (**Fig 1F and 1G**), based on the percent of the epithelium staining positive for each marker through net analysis (**Fig 1H and 1I**).

**Fig 2:**
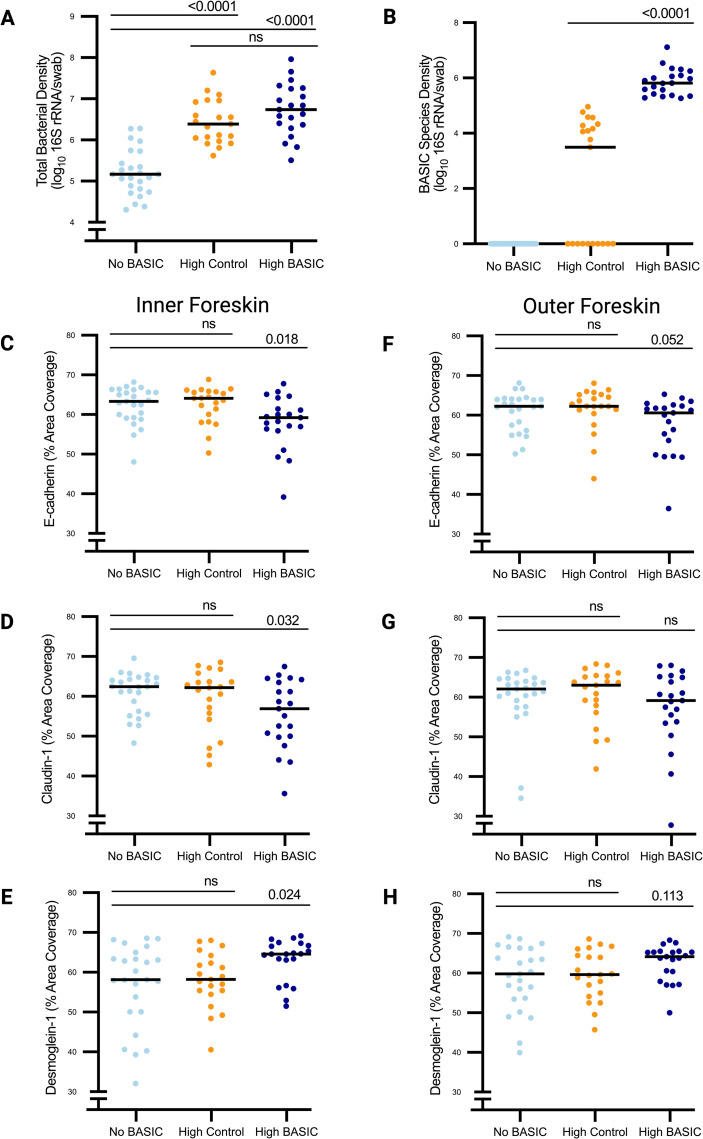
BASIC species are associated with altered expression of epithelial junction proteins in the inner foreskin. Inner and outer foreskin tissue samples were divided into 3 groups; No BASIC (n = 25), High Control (n = 21), and High BASIC (n = 21). Total bacterial density (**A**, 16S rRNA qPCR) and BASIC species density (**B**, 16S rRNA sequencing and qPCR) was determined from penile swabs. Relative expression of epithelial junction proteins E-cadherin (C, F), claudin-1 (D, G), and Desmoglein-1 (**E, H**) in foreskin tissues was determined by quantitative immunofluorescent microscopy on tissues from both the inner (C, D, E) and outer (F, G, H) aspects of the foreskin. Statistical comparisons were made using the Wilcoxon rank-sum test, lines shown for median in each group.

Junction protein expression was compared between participants grouped by the combined bacterial absolute abundance of BASIC species or control taxa. These groupings are described in detail in the methods section. Briefly, the “High BASIC” group contains participants with a high absolute abundance of BASIC species but not high control taxa. The “High Control” group contains participants with a high absolute abundance of *Staphylococcus*, *Corynebacterium*, *Negativicoccus*, and *Helococcus*, but not high BASIC species. The “No BASIC” group contains participants with no detected BASIC species and low levels of control taxa. Participants in the High Control and High BASIC groups had similar total bacterial load (6.44 and 6.74 log_10_ 16S rRNA/swab respectively, p = 0.22,), while participants in the No BASIC group had lower total bacterial load (5.20 log_10_ 16S rRNA/swab, p < 0.0001, **Fig 2A**) compared to both High BASIC and High Control. While total bacterial load was similar between High BASIC and High Control groups, participants in the High BASIC group had a higher density of BASIC species (5.86 vs. 2.24 log_10_ 16S rRNA/swab, respectively, p < 0.0001 **Fig 2B**). Median relative bacterial abundances of BASIC species and control taxa for each group are shown in S2 Table.

In the inner foreskin, participants in the High BASIC group had lower expression of epithelial junction proteins E-cadherin (59.2 vs. 63.3% area, p = 0.018) and claudin-1 (56.9 vs. 62.4% area, p = 0.032), but higher expression of desmoglein-1 (64.6 vs. 58.2% area, p = 0.024), compared to the No BASIC group (Fig 2C–E). These differences remained after controlling for previous antimicrobial treatments (adjusted p-values: E-cadherin p = 0.016, claudin-1 p = 0.037, desmoglein-1 p = 0.018). Similar trends and effect sizes were observed in the “High BASIC” group compared to the “No BASIC” group for all three proteins even when participants who received topical antimicrobials were excluded. These effect sizes were -3.1, -5.5, and + 12.0% area for E-cadherin, claudin-1, and desmoglein-1 respectively (S1 Appendix), compared to -4.1, -5.3, and + 6.4% area shown in Fig 2C–E. In the outer foreskin, group effects mirrored the inner foreskin, but differences between groups were smaller in magnitude and did not reach statistical significance (Fig 2F–H).

### *Prevotella bivia* can cleave E-cadherin

**Fig 3:**
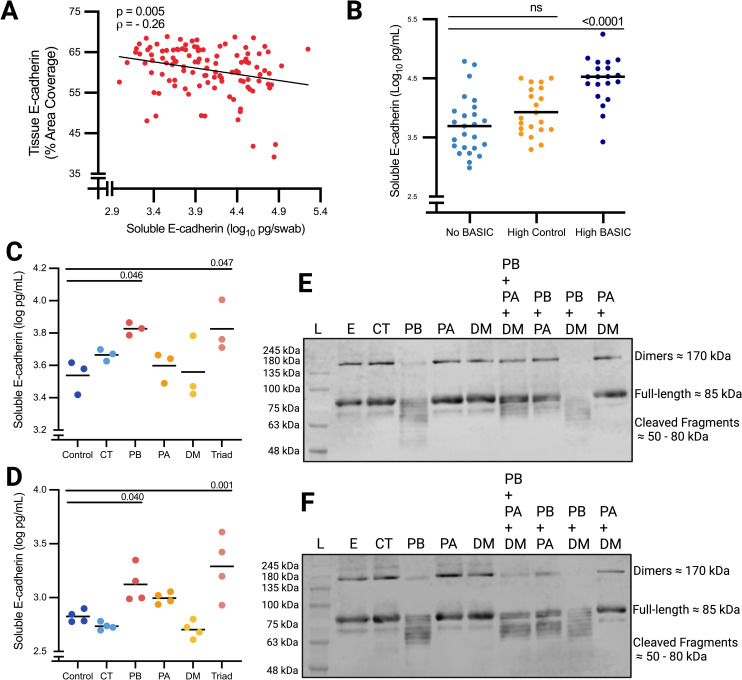
*Prevotella bivia* can cleave E-cadherin. Soluble E-cadherin from all participants was quantified in penile swabs and negatively correlated with tissue E-cadherin (immunofluorescence) (**A**, n = 116, Spearman’s correlation). Soluble E-cadherin was compared between No BASIC (n = 25), High BASIC (n = 21), and High Control (n = 21) groups **(B)**. *In* vitro, BASIC species were grown in monocultures or together then added as live bacteria (**C**) or concentrated conditioned media (**D**) to primary human foreskin epithelial cells; soluble E-cadherin release was quantified by ELISA. Data represent means of three independent experiments for (**C**) and individual technical replicates for **(D)**. Live bacteria (**E**) or concentrated conditioned media (**F**) were also added directly to recombinant human E-cadherin to assess cleavage. L = ladder, E = E-cadherin alone, CT = *Corynebacterium tuberculostearicum*, PB = *Prevotella bivia*, PA = *Peptostreptococcus anaerobius*, DM = *Dialister Micraerophilus*. Lines represent means in all figures. Student’s t-test **(B)**, or ANOVA with Tukey’s test was used to compare groups **(C, D)**, both α = 0.05.

Soluble E-cadherin was quantified from penile swabs to measure E-cadherin proteolytic cleavage. Soluble E-cadherin correlated negatively with tissue E-cadherin (**Fig 3A**, p = 0.005), and participants in the High BASIC group had elevated soluble E-cadherin compared to the No BASIC group (**Fig 3B**, 4.36 vs 3.71 log_10_ pg/mL, p < 0.0001). This difference remained after controlling for previous antimicrobial treatments (adjusted p < 0.0001). The same correlation was observed when excluding participants who received topical treatments (S2A Appendix). The effect size of the increase in soluble E-cadherin in the “High BASIC” group compared to the “No BASIC” group was also similar in this sensitivity analysis (+0.66 log_10_ pg/mL, S2B Appendix) compared to that shown in **Fig 3B** (+0.75 log_10_ pg/mL).

BASIC species were further tested for E-cadherin cleavage *in vitro*. Soluble E-cadherin release was measured from primary foreskin epithelial cells after incubation with penile isolates of BASIC species or the control species *Corynebacterium tuberculostearicum*. Soluble E-cadherin was elevated after exposure to *Prevotella bivia,* alone (3.83 vs 3.54 log_10_ pg/mL, adjusted p < 0.05) or in combination with other BASIC species (3.83 vs 3.54 log_10_ pg/mL, adjusted p < 0.05) (**Fig 3C**), compared to media alone. Soluble E-cadherin was also elevated after exposure to conditioned media from *Prevotella bivia,* alone (3.12 vs 2.83 log_10_ pg/mL, adjusted p = 0.04) or in combination with other BASIC species (3.29 vs 2.83 log_10_ pg/mL, adjusted p = 0.001, **Fig 3D**). No changes in soluble E-cadherin were observed after incubation of epithelial cells with live bacteria or conditioned media for *Corynebacterium tuberculostearicum*, *Peptostreptococcus anaerobius*, or *Dialister micraerophilus* alone.

After incubation with recombinant human E-cadherin, Western blotting demonstrated that *Prevotella bivia* was able to directly cleave full length E-cadherin (~85 kDa) and E-cadherin dimers (~170 kDa) into a series of smaller fragments of roughly 50–80 kDa (**Fig 3E**). This same effect was observed in any combination of bacteria that included *Prevotella Bivia*, but not with the control species *Corynebacterium tuberculostearicum* or with the BASIC species *Peptostreptococcus anaerobius* or *Dialister micraerophilus* alone. Cleavage was also observed when conditioned media was added to recombinant E-cadherin instead of live bacteria (**Fig 3F**).

Tissue and soluble E-cadherin were then compared between RCT participants grouped by absolute abundance of *Prevotella bivia*: High *Prevotella bivia* (n = 23), High Control (n = 19), and No *Prevotella bivia* (n = 54). Participants in the High *Prevotella bivia* group had lower E-cadherin expression in the inner (S3A Appendix 58.9 vs. 61.8% area, p = 0.031) and outer (S3B Appendix 58.0 vs. 61.3% area, p = 0.011) foreskin, but higher soluble E-cadherin (S3C Appendix 4.37 vs. 3.89 log_10_ pg/mL, p = 0.0002), compared to the No *Prevotella bivia* group.

### BASIC species are associated with a thicker epithelium and increased keratinocyte proliferation

**Fig 4:**
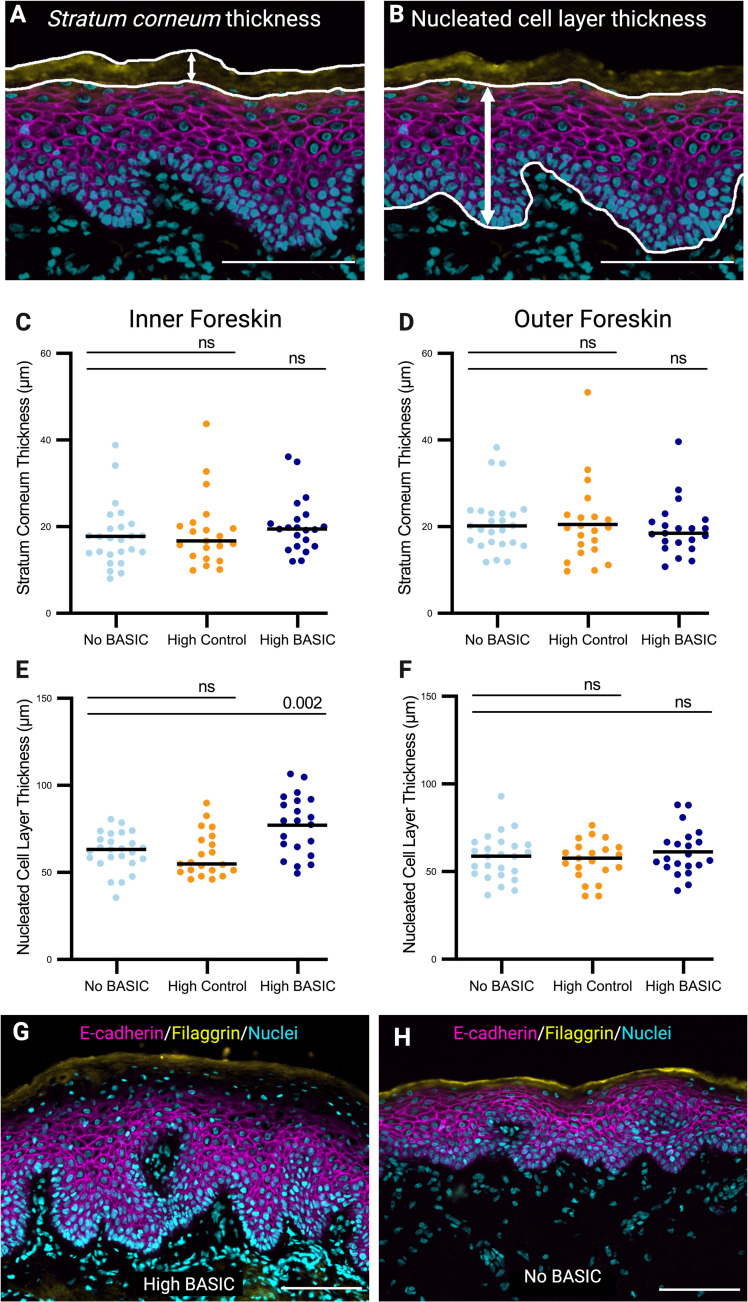
BASIC species are associated with thicker nucleated cell layers, but not *stratum corneum* thickness. The edges of the *stratum corneum* (**A**) and nucleated epithelium (**B**) were manually traced from immunofluorescence images and the mean distance between traced lines was quantified for the *stratum corneum* (**C–D**) and nucleated epithelial layers **(E–F)**. Mean thicknesses were compared between men in the No BASIC (n = 25) group and men in the High BASIC (n = 21) and High Control (n = 21) groups (Student’s t-test, lines represent means). Representative inner foreskin images from High BASIC and No BASIC groups shown in **G** and **H**, respectively (scale bars 100 µm). Representative images were chosen from samples near the median thickness in each group. Brightness and contrast have been enhanced from the original images for visualization purposes.

Epithelial thickness was measured for two epithelial layers: the *stratum corneum* (**Fig 4A**) and the nucleated epithelium (**Fig 4B**). No differences were observed in *stratum corneum* thickness between groups in the inner (**Fig 4C**) or outer (**Fig 4D**) foreskin. However, the nucleated inner foreskin epithelium was thicker for participants in the High BASIC group compared to the No BASIC group (77.10 vs. 62.07 µm, p < 0.001) (**Fig 4E**). This difference remained after controlling for previous antimicrobial treatments (adjusted p < 0.001). Furthermore, a similar trend was observed when men receiving topical treatments were excluded from the analysis (S2E Appendix). The effect size of the increase in nucleated inner foreskin epithelial thickness in the “High BASIC” group compared to the “No BASIC” group was also similar in this sensitivity analysis (+12.43 µm) compared to that shown in **Fig 4E** (+15.03 µm). Representative inner foreskin images from High BASIC and No BASIC groups are shown in **Fig 4G** and **4H** respectively.

**Fig 5:**
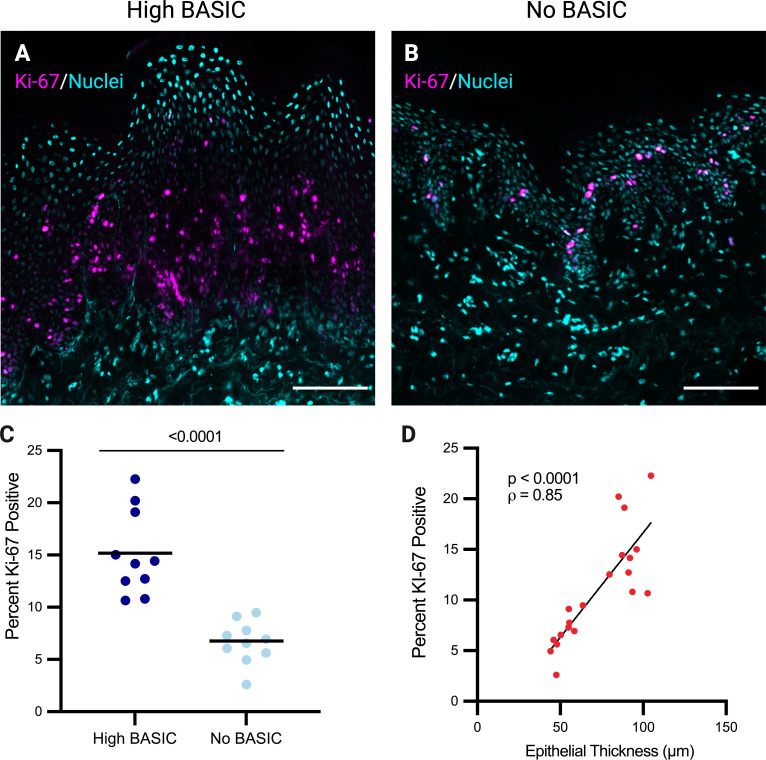
BASIC species are associated with increased keratinocyte proliferation. Ki-67 expression was quantified by immunofluorescence in a subset of participants with High BASIC (n = 10) or No BASIC (n = 10) species (representative images in **A** and **B**). Mean percentage of Ki-67 positive cells was compared between groups (**C**, Student’s t-test, lines represent means). Percent Ki-67 + cells correlated with thickness of the nucleated cell layers of the epithelium (**D**, Spearman’s correlation). Brightness and contrast have been enhanced from the original images for visualization purposes.

To determine if the increased nucleated epithelial thickness in High BASIC participants was due to elevated keratinocyte proliferation, epithelial expression of the cell proliferation marker Ki-67 was quantified in n = 10 inner foreskin samples from each of the High BASIC and no BASIC groups (**Fig 5A** **and** **5B**). Participants in the High BASIC group had a higher proportion of proliferating cells compared to the No BASIC group (15.19 vs. 6.64% Ki-67 + , p < 0.0001) (**Fig 5C**). Ki-67 expression correlated with epithelial thickness (**Fig 5D**, p = 0.005).

## Discussion

In this study, we provide evidence that the same bacteria associated with penile HIV acquisition alter important characteristics of the foreskin epithelium. A high absolute abundance of BASIC species was associated with reduced expression of epithelial junction proteins E-cadherin and claudin-1, but increased desmoglein-1 expression. We also observed a positive correlation between BASIC species absolute abundance and soluble E-cadherin on the penile surface. Soluble E-cadherin is not synthesized directly, but is instead a product of ectodomain cleavage of E-cadherin from adherens junctions, suggesting that BASIC species cause E-cadherin proteolysis [15]. *In vitro*, the BASIC species *Prevotella bivia* (and its conditioned media), but not *Peptostreptococcus anaerobius* or *Dialister micraerophilus*, were able to directly cleave E-cadherin and stimulate soluble E-cadherin release from primary human foreskin epithelial cells.

A wide variety of host and bacterial proteases can cleave E-cadherin [16]. Host epithelial cell proteases known to cleave epithelial junction proteins, such as those belonging to the ADAM protein family [17], are important for regulating cell-cell adhesion under physiological conditions [17], but can be targeted and upregulated by a diverse range of bacterial effector proteins [18–21]. Bacteria can also express their own proteases to directly cleave epithelial junction proteins, as has been shown for *Helicobacter pylori* [18], *Escherichia coli* [22], *Shigella flexneri* [23], *Campylobacter jejuni* [24,25], and *Porphyromonas gingivalis* [26], among others. Many proteases have been characterized in the genus *Prevotella* [27], although none, to our knowledge, have been previously directly linked to cleavage of an epithelial junction protein.

Studies in the female genital tract have demonstrated that HIV virions penetrate tissue more effectively in areas with reduced tissue E-cadherin [28], suggesting loss of E-cadherin may be a mechanism by which BASIC species increase penile HIV acquisition. Elevated soluble E-cadherin in the female genital tract is associated with HIV risk [29], as are the anaerobic bacteria seen in bacterial vaginosis [30]. One study recently found compromised epithelial barrier integrity, including reduced intact E-cadherin staining in ectocervical tissues from HIV+ female sex workers [31].

However, while E-cadherin is important for cell-cell attachment and adherens junction formation, tight junctions canonically control diffusion across the epithelium. We observed that BASIC species were associated with decreased expression of the tight junction protein claudin-1.

E-cadherin is required for tight junction formation [32]; therefore, disruption of E-cadherin may also compromise tight junctions. It may also be the case that *Prevotella bivia* or other BASIC species secrete proteases which can directly cleave tight junction proteins. Further *in vitro* studies are required to elucidate the connections between BASIC species, epithelial integrity, and HIV penetration.

BASIC species were also unexpectedly associated with increased thickness of the nucleated inner foreskin epithelium and increased expression of the desmosome protein desmoglein-1, both of which would not intuitively be expected to increase HIV acquisition. There are a few possible explanations for the observed increase in thickness; firstly, keratinocyte proliferation driving epithelial thickening often accompanies ongoing epithelial inflammation, and several proinflammatory cytokines previously linked to increased HIV acquisition can stimulate basal epithelial cell proliferation (IL-1a and GC-CSF) [33,34]. Furthermore, the thickening of the *stratum spinosum* layer of the epithelium (acanthosis), is commonly seen in inflammatory skin conditions, such as atopic dermatitis [35] (eczema) and UV damage [36]. Therefore, it is reasonable to speculate that this proliferation and resultant increase in epithelial thickness could be the result of chronic inflammation from long-term colonization of BASIC species. Importantly, a similar finding has been observed in the female genital tract, where anaerobic bacteria were found to be associated with cell proliferation and inflammation [37]. Another possibility is that keratinocyte proliferation is driven by the loss of cell-cell contact inhibition; intact E-cadherin homophilic binding induces growth inhibitory signals that limit and regulate cell proliferation [38]. Cleavage of E-cadherin by proteases—induced or expressed by BASIC species—may disrupt contact inhibition and promote keratinocyte proliferation. The upregulation of desmoglein-1 may also be due to E-cadherin cleavage. In E-cadherin knockout mice, adherens junctions are lost but desmosomes are left intact and functional [39,40]. Desmosomal cadherins, including desmoglein-1, are upregulated in the nucleated epithelium, which may be a compensatory effect to maintain tissue integrity after the loss of E-cadherin [40]. In these same mice, substantial acanthosis was observed in the nucleated epithelium, thought to be the result of altered keratinocyte differentiation [39] or hyperproliferation [40]. More *in vitro* research is needed to determine if these processes are induced in the foreskin as a response to colonization with BASIC species.

A strength of this study is that it provides *in vivo* data from a relatively large cohort of adult males. However, the data are necessarily cross-sectional since MMC is only performed once for each participant, imposing several limitations on the study. First, while BASIC species were associated with epithelial changes, it is not possible to establish causality in this observational study. Although differences were not associated with the total bacterial load, other (unquantified) bacteria or unmeasured confounders may contribute to changes in the epithelium. Additional experiments will be required to confirm a causal relationship between BASIC species and alterations to the epithelium, as well as to determine the individual effects of each co-occurring BASIC species [4]. An *in vitro* model that recapitulates the dry, stratified epithelial structure and low-oxygen environment of the foreskin would be one approach to establish causality and determine the effects of individual BASIC species on the foreskin epithelium.

Another limitation of this study is that the antimicrobial treatments assigned during the RCT impose a potential confounding variable on our results. In the RCT, topical metronidazole and clindamycin significantly reduced BASIC species absolute abundance and soluble E-cadherin levels. Topical metronidazole was also associated with higher tissue E-cadherin than no treatment group [14]. The most likely explanation for these observations is through the direct reduction in BASIC species by these antimicrobial treatments. To this end, we performed multivariate analyses exploring the effects of both BASIC species absolute abundance and antimicrobial treatments on epithelial characteristics. From these analyses, we saw that BASIC species remained associated with epithelial differences, and that treatment group was not significantly associated with epithelial characteristics (when controlling for BASIC absolute abundance). We can therefore reasonably attribute the observed differences in epithelial characteristics to differential BASIC species absolute abundance. This is further evidenced by similar trends and effect sizes being observed for all measured outcomes in a supplemental analysis limited to individuals who did not previously apply topical antimicrobial treatments. Of note, we were not able to examine the longitudinal effect of antimicrobial treatments on tissue parameters as tissues were only collected once, at RCT conclusion.

Overall, this study demonstrates for the first time that HIV-associated anaerobic bacteria are associated with substantially altered characteristics of the inner foreskin epithelium, an important site of HIV acquisition [41,42]. We also provide evidence that *Prevotella bivia* secretes at least one protease able to directly cleave the adherens junction protein E-cadherin. Reduced tissue E-cadherin has been previously associated with HIV penetration, and it is likely that BASIC species are inducing epithelial changes that render the foreskin more permissive to HIV and other pathogens. Targeting these bacteria therefore holds promise as a preventative therapy for a wide range of sexually transmitted infections.

## Methods

### Ethics statement

The Randomized Controlled Trial was reviewed and approved by the Uganda Virus Research Institute Research and Ethics Committee (GC/127/17/07/613), the Uganda National Council for Science and Technology (HS2299), the Uganda National Drug Authority (clinical trial certificate #CTA0041), the University of Toronto Research Ethics Board (RIS #35254), and the Western University Human Ethics Research Board (Protocol 111441). All participants provided written informed consent. Microbiome analysis was performed at George Washington University and was determined to be non-human subjects research by the Institutional Review Board (00000169). Foreskin tissues for keratinocyte isolation were collected under a research protocol reviewed and approved by the Western University Human Ethics Research Board (Protocol 113008). Written informed consent was obtained from all participants or their sub-decision maker if the participant was under the age of 14 years. For participants between the ages of 9–13 years, assent was obtained in addition to sub-decision maker consent.

### Study population

Biological samples were collected from 116 HIV-negative uncircumcised males enrolled in a randomized controlled trial (RCT) performed in Uganda in 2019, exploring the effect of antimicrobial agents on the penile microbiota, immunology, and HIV susceptibility [43]. The primary endpoint of this study was *ex vivo* HIV entry into inner foreskin derived CD4 + T cells. The results from this clinical trial have been previously described [14].

### Biological sample collection and processing

Detailed methods for the collection of penile swabs and tissues have been previously described [14]. Briefly, inner foreskin swabs were collected by a study clinician immediately prior to surgical cleaning and MMC. Swab eluant was aliquoted and stored at -80^o^C. Following MMC, visible blood vessels and clots were excised from foreskin tissues. Inner and outer foreskin sections of 0.25 cm^2^ were placed into optimal cutting temperature (OCT) compound and stored at -80˚C.

### Immunofluorescence staining

Frozen foreskin tissue sections (10 µm) were fixed for 10 minutes in 3.7% formaldehyde in 0.1 M PIPES buffer, pH 6.8. Sections were blocked with 10% normal donkey serum (Avantor), 0.1% Triton X-100 (Sigma-Aldrich) and 0.1% Sodium Azide (Sigma-Aldrich) in PBS. Antibodies used are listed in S2 Table. Tissue sections were incubated with primary antibodies at 37˚C for 1 hour and with secondary antibodies at 25˚C for 30 minutes. Sections were washed with PBS between incubations. Fluoromount-G Mounting Medium with DAPI (ThermoFisher) was used to visualize cell nuclei.

### Immunofluorescence microscopy

Tiled images of whole foreskin tissue sections were imaged (200×) using a DM5500B fluorescence microscope (Leica) and exported as TIF files. Apical and basal epidermal edges were manually identified for analysis. All analyses were performed on full tissue sections (as opposed to fields of view). Tissue folds and artifacts were manually excluded.

### Epithelial junction protein expression and thickness measurements

Junction protein expression and epithelial thicknesses were determined using methods similar to those previously described [44]. Briefly, relative fluorescence was used to define a net-like structure of epithelial junction protein expression for each protein (E-cadherin, claudin-1, and desmoglein-1), which is influenced by both the brightness and distribution of positive staining. Epithelial junction protein expression was quantified as the percentage of the epithelium covered by the net structure (% area).

Staining for the *stratum corneum* protein filaggrin was used to aid in manual identification of the boundary between the *stratum corneum* and the nucleated epithelium. Apical and basal edges of (i) the *stratum corneum* and (ii) nucleated epithelium were manually traced, with thicknesses measured using the distance transform function in CellProfiler (Version 2.2.0 and 3.1.8) [45]. This function determines the minimum distance of each pixel on the apical edge to the basal edge and vice versa, which were averaged to cell layer thicknesses. All tracing and image analyses were performed by an investigator blinded to treatment group and microbiome data.

### Quantification of epithelial cell proliferation

The epithelium was manually segmented from full-tissue images. Pixel-based segmentation was then used to identify cell nuclei using ImageJ software (version 2.1.0/1.53c) as previously described [46]. Ki-67 positive cells were counted manually and expressed as a percentage of total epithelial cells.

### Soluble E-cadherin quantification

An electrochemiluminescent-based detection system (MesoScale Discovery) was used to quantify soluble E-cadherin from penile swabs [47]. Samples were run in duplicate, and any sample with a coefficient of variation >30%, or that exceeded the upper limit of quantification, were re‐run. Concentrations below the threshold of detection were assigned as the lower limit of detection, which was calculated to be 29.97 pg/mL, derived from the mean of all standard curves. A soluble E-cadherin ELISA kit (Abcam) was similarly used to quantify soluble E-cadherin from *in vitro* experiments.

### Bacterial quantification

Detailed methods for quantifying penile bacterial species have been previously described [14]. Total DNA was extracted from coronal sulcus swab eluent using a combination of chemical and enzymatic lysis. Penile microbiota were characterized by 16S rRNA gene–based amplicon sequencing (V3V4) and by broad-range real-time PCR (V3V4). Species-level classification for select genera was performed using an in-house Bayesian classifier trained by a curated training set. Using the resultant sequencing and qPCR data, absolute abundance of each penile taxon was calculated as the product of total bacterial load and proportional abundance of the taxa [48,49].

### In vitro assays

Penile BASIC species isolates were grown on Columbia blood agar plates, then incubated anaerobically at 37˚ C for 24 hours in Columbia broth. *Corynebacterium tuberculostearicum* was grown similarly at atmospheric oxygen. The 600 nm absorbance of cultures was measured to normalize inocula to an OD of 0.06. Bacteria (4 µ L/well) were added to a 96-well plate with pre-reduced Columbia broth (14 µ L) and recombinant human E-cadherin (12 µ L of 0.01 mg/mL, Sino Biologicals). Conditioned media was filter sterilized and concentrated with Amicon ultra centrifugal filters (10 kDa MW cutoff), with 18 µ L/well added to recombinant E-cadherin. Samples were incubated anaerobically for 12 (live bacteria) or 8 (conditioned media) hours at 37˚ C before collection.

### Western blotting

Samples were added to Tris-glycine sodium dodecyl sulfate (SDS) sample buffer (Invitrogen), separated by SDS-polyacrylamide gel electrophoresis, and transferred to 0.2 µm polyvinylidene fluoride membranes (ThermoFisher). Samples were blocked with LI-COR Intercept blocking buffer for 1 hour, incubated with mouse anti-human E-cadherin antibody (Santa Cruz Biotech, 1:200) overnight, and incubated with goat anti-mouse secondary antibody conjugated to IRDye 800CW (LI-COR, 1:10000) for 1 hour. Blots were imaged with a LI-COR Odyssey CLx Imager.

### Cell culture

Primary human foreskin keratinocytes were isolated from tissues donated after MMC as previously described [50]. Keratinocytes were transduced with replication incompetent retroviral vector (pLXSN16E6E7) expressing HPV16 E6 and E7 proteins. Cells were cultured in T75 culture flasks with DermaCult keratinocyte expansion media (200 nM hydrocortisone), then grown to confluence in 24-well plates. Cells were washed with PBS and received new media prior to adding bacteria. Bacterial inocula were normalized to a 600 nm OD reading of 0.6, and 25 µl was added with 225 µ L fresh media to epithelial cells. Cells were co-cultured with bacteria for 12 hours in an anerobic box before media collection.

### Data analysis

Analyses were pre-defined, focusing on the combined absolute abundance of BASIC species [4], specifically *Peptostreptococcus anaerobius, Prevotella bivia, Prevotella disiens, Dialister propionicifaciens, Dialister micraerophilus,* and a genetic near neighbour of *Dialister succinatiphilus*. These BASIC species are associated with HIV seroconversion, pro-inflammatory cytokines, and increased density of HIV target cells in the foreskin [4]. Control taxa included: (i) two major taxa that increase in absolute abundance after MMC (*Staphylococcus* and *Corynebacterium*) [51] and (ii) two anaerobic taxa that decrease in absolute abundance after MMC but are not associated with HIV seroconversion (*Negativicoccus* and *Helococcus*) [4].

For epithelial integrity analyses, participants were divided into three mutually exclusive groups: “High BASIC” (n = 21), “High Control” (n = 21), and “No BASIC” (n = 25). The “High BASIC” group was defined as individuals in the top quartile (5.26 log_10_ 16S RNA copies/swab and above) of BASIC species absolute abundance but not the top quartile of control taxa absolute abundance. The “High Control” group was defined as individuals in the top quartile (5.40 log_10_ 16S RNA copies/swab and above) of control taxa absolute abundance but not the top quartile of BASIC species absolute abundance. The “No BASIC” group was defined as individuals with no detected BASIC species without being in the top quartile of control taxa absolute abundance. For the S3 Appendix *Prevotella bivia* analyses, groups were defined similarly, with *Prevotella bivia* absolute abundance replacing BASIC species absolute abundance. Total bacterial load and BASIC species absolute abundance were compared between the three groups using one-way ANOVA and Tukey’s test (α=0.05). Subsequent pairwise comparisons were made between the “No BASIC” group and the other two groups using Student’s t-test or Wilcoxon’s rank-sum test, α=0.05. Correlations were performed using Spearman’s correlation, α=0.05. To determine if previous antimicrobials treatments from the RCT had any effect on observed associations, we controlled for RCT treatment assignment in all analyses using multivariate linear regression. One-way ANOVA and Tukey’s test (α=0.05) was used to compare groups for *in vitro* experiments. GraphPad Prism (Version 9) was used for statistical analyses and to generate graphs. Figures were assembled using BioRender.com.

## Supporting information

S1 Table Antibodies used for immunofluorescence. (DOCX)

S2 Table Median relative abundance of bacterial groupings. (DOCX)

S1 Appendix Epithelial junction protein expression in no treatment groups. Epithelial junction protein expression was quantified in immunofluorescence images from participant foreskin tissue samples from the no treatment (blue) and oral tinidazole (red) treatment groups. Relative expression of epithelial junction proteins E-cadherin (**A B**), claudin-1 (**C, D**), and desmoglein-1 (**E, F**) was measured in tissues from both the inner (A, C, E) and outer (B, D, F) aspects of the foreskin. Comparisons were made between bacterial groupings as described in the methods section; No BASIC (n = 4), High Control (n = 7), and High BASIC (n = 13).
(DOCX)

S2 Appendix Soluble E-cadherin and epithelial thickness in no treatment groups. Soluble E-cadherin and epithelial thickness were measured from participant samples from the control (blue) and oral tinidazole (red) treatment groups. Soluble E-cadherin quantified using multiplexed ELISA from penile swabs inversely correlated with tissue E-cadherin measured from quantitative immunofluorescence microscopy (**A**, Spearman’s correlation, n = 25). Soluble E-cadherin was also compared between bacterial treatment groups (**B**). *Stratum corneum* thickness (**C**, **D**) and epithelial thickness (**E**, **F**) were measured from immunofluorescence images from both the inner (**C**, **E**) and outer (**D**, **F**) aspects of the foreskin. Comparisons were made between bacterial groupings as described in the methods section; No BASIC (n = 4), High Control (n = 7), and High BASIC (n = 13).
(DOCX)

S3 Appendix E-cadherin expression by *Prevotella bivia* absolute abundance. Absolute abundance of *Prevotella bivia* (16S rRNA sequencing and qPCR) was determined from penile swabs. Inner and outer foreskin tissue samples were divided into 3 groups; No *Prevotella bivia* (n = 54), High Control (n = 19), and High *Prevotella bivia* (n = 23). Relative expression of the epithelial junction protein E-cadherin in inner (**A**) and outer (**B**) foreskin tissues was determined by quantitative immunofluorescent microscopy. Soluble E-cadherin was measured using ELISA (**C**). Student’s T test, α = 0.05. Lines shown for mean in each group.
(DOCX)

S4 Appendix Raw Data. (XLSX)
