# Supplementary material for: HIV-associated penile anaerobes disrupt epithelial barrier integrity
Source: PLoS Pathog. 2025 Apr 17;21(4):e1013094. doi: 10.1371/journal.ppat.1013094 (PMC12040277; doi:10.1371/journal.ppat.1013094)
Supplement: S1 Table — (DOCX) [file ppat.1013094.s001.docx]

**S1 Table.** **Antibodies used for immunofluorescence**

| **Target** | **1^o^/ 2^o^** | **Clone** | **Supplier** | **Host Species** | **Dilution** | **Fluorophore** |
| --- | --- | --- | --- | --- | --- | --- |
| E-cadherin | 1^o^ | 36/E-Cadherin | BD | Mouse | 1:50 | None |
| Claudin-1 | 1^o^ | Polyclonal | Abcam | Rabbit | 1:200 | None |
| Desmoglein-1 | 1^o^ | 27B2 | Abcam | Mouse | 1:100 | None |
| Filaggrin | 1^o^ | AKH1 | Santa Cruz | Mouse | 1:50 | Alexa Fluor 488 |
| Ki-67 | 1^o^ | SP6 | Fisher | Rabbit | 1:100 | None |
| Mouse IgG^†^ | 2^o^ | Polyclonal | Fisher | Donkey | 1:400 | Alexa Fluor 647 |
| Rabbit IgG^†^ | 2^o^ | Polyclonal | Fisher | Donkey | 1:400 | Alexa Fluor 647 |

^†^ Binds both heavy and light IgG chains (H + L)
