# Supplementary material for: HIV-associated penile anaerobes disrupt epithelial barrier integrity
Source: PLoS Pathog. 2025 Apr 17;21(4):e1013094. doi: 10.1371/journal.ppat.1013094 (PMC12040277; doi:10.1371/journal.ppat.1013094)
Supplement: S2 Table — (DOCX) [file ppat.1013094.s002.docx]

**S2 Table.** **Median relative abundance of bacterial groupings**

| **Group** | **BASIC species median relative abundance** | **Control taxa median relative abundance** | **Other taxa median relative abundance** |
| --- | --- | --- | --- |
| No BASIC (n=25) | 0.000 % | 35.9 % | 64.1 % |
| High Control (n=21) | 0.121 % | 50.0 % | 49.8 % |
| High BASIC (n=21) | 22.2 % | 0.685 % | 77.8 % |
| All Participants (n=116) | 1.28 % | 10.8 % | 88.0 % |
