# Supplementary material for: HIV-associated penile anaerobes disrupt epithelial barrier integrity
Source: PLoS Pathog. 2025 Apr 17;21(4):e1013094. doi: 10.1371/journal.ppat.1013094 (PMC12040277; doi:10.1371/journal.ppat.1013094)
Supplement: S1 Appendix — Epithelial junction protein expression was quantified in immunofluorescence images from participant foreskin tissue samples from the no treatment (blue) and oral tinidazole (red) treatment groups. Relative expression of epithelial junction proteins E-cadherin (A B), claudin-1 (C, D), and desmoglein-1 (E, F) was measured in tissues from both the inner (A, C, E) and outer (B, D, F) aspects of the foreskin. Comparisons were made between bacterial groupings as described in the methods section; No BASIC (n = 4), High Control (n = 7), and High BASIC (n = 13). (DOCX) [file ppat.1013094.s003.docx]

**S1 Appendix.** **Epithelial junction protein expression in no treatment groups.** Epithelial junction protein expression was quantified in immunofluorescence images from participant foreskin tissue samples from the no treatment (blue) and oral tinidazole (red) treatment groups. Relative expression of epithelial junction proteins E-cadherin (**A B**), claudin-1 (**C, D**), and desmoglein-1 (**E, F**) was measured in tissues from both the inner (A, C, E) and outer (B, D, F) aspects of the foreskin. Comparisons were made between bacterial groupings as described in the methods section; No BASIC (n = 4), High Control (n = 7), and High BASIC (n = 13).
