# Supplementary material for: HIV-associated penile anaerobes disrupt epithelial barrier integrity
Source: PLoS Pathog. 2025 Apr 17;21(4):e1013094. doi: 10.1371/journal.ppat.1013094 (PMC12040277; doi:10.1371/journal.ppat.1013094)
Supplement: S2 Appendix — Soluble E-cadherin and epithelial thickness were measured from participant samples from the control (blue) and oral tinidazole (red) treatment groups. Soluble E-cadherin quantified using multiplexed ELISA from penile swabs inversely correlated with tissue E-cadherin measured from quantitative immunofluorescence microscopy (A, Spearman’s correlation, n = 25). Soluble E-cadherin was also compared between bacterial treatment groups (B). Stratum corneum thickness (C, D) and epithelial thickness (E, F) were measured from immunofluorescence images from both the inner (C, E) and outer (D, F) aspects of the foreskin. Comparisons were made between bacterial groupings as described in the methods section; No BASIC (n = 4), High Control (n = 7), and High BASIC (n = 13). (DOCX) [file ppat.1013094.s004.docx]

**
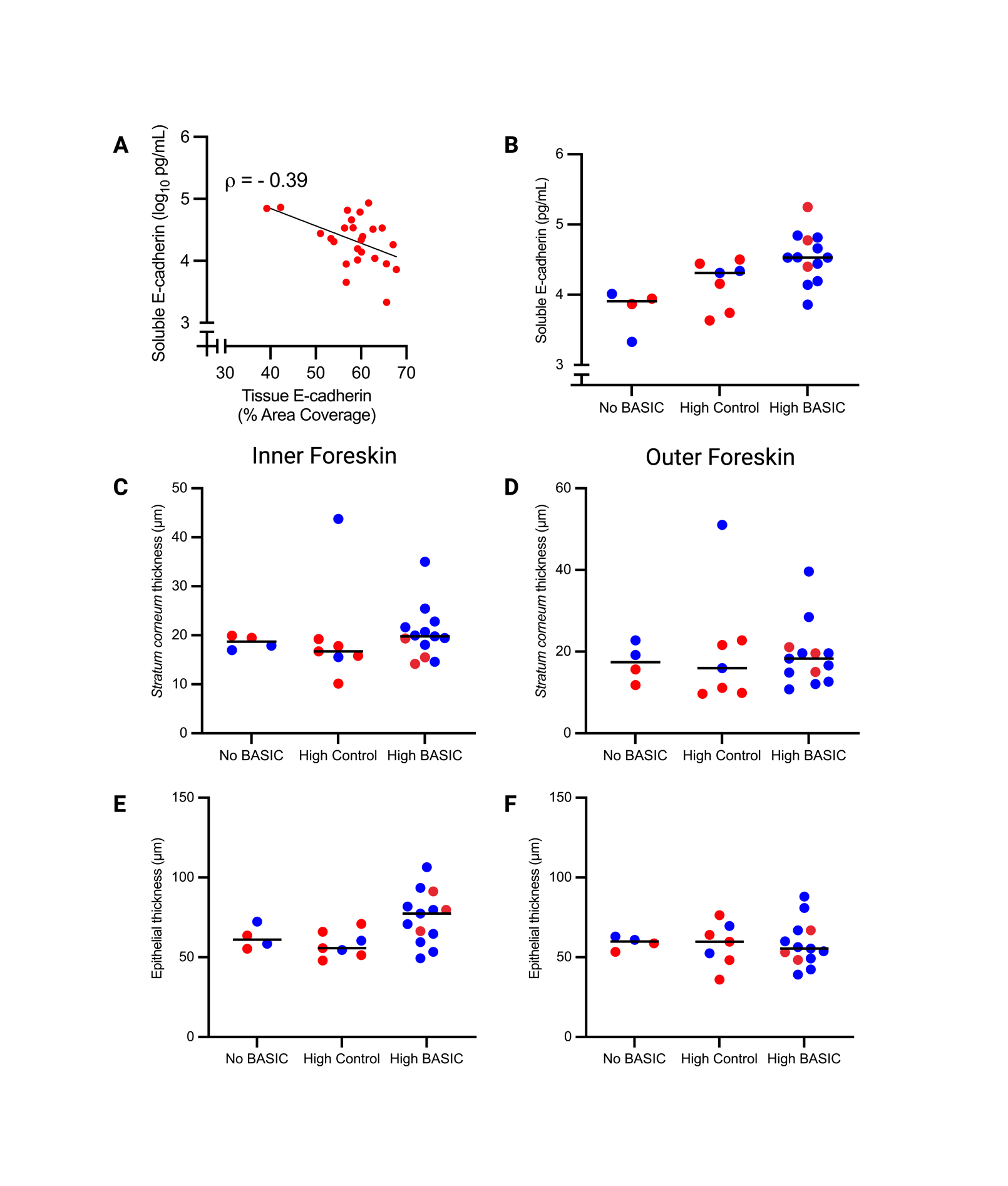
**

**S2 Appendix. Soluble E-cadherin and epithelial thickness in no treatment groups**. Soluble E-cadherin and epithelial thickness were measured from participant samples from the no treatment (blue) and oral tinidazole (red) treatment groups. Soluble E-cadherin quantified using multiplexed ELISA from penile swabs inversely correlated with tissue E-cadherin measured from quantitative immunofluorescence microscopy (**A**, Spearman’s correlation, n=25). Soluble E-cadherin was also compared between bacterial treatment groups (**B**). *Stratum corneum* thickness (**C**, **D**) and epithelial thickness (**E**, **F**) were measured from immunofluorescence images from both the inner (**C**, **E**) and outer (**D**, **F**) aspects of the foreskin. Comparisons were made between bacterial groupings as described in the methods section; No BASIC (n=4), High Control (n=7), and High BASIC (n=13).
