# Supplementary material for: HIV-associated penile anaerobes disrupt epithelial barrier integrity
Source: PLoS Pathog. 2025 Apr 17;21(4):e1013094. doi: 10.1371/journal.ppat.1013094 (PMC12040277; doi:10.1371/journal.ppat.1013094)
Supplement: S3 Appendix — Absolute abundance of Prevotella bivia (16S rRNA sequencing and qPCR) was determined from penile swabs. Inner and outer foreskin tissue samples were divided into 3 groups; No Prevotella bivia (n = 54), High Control (n = 19), and High Prevotella bivia (n = 23). Relative expression of the epithelial junction protein E-cadherin in inner (A) and outer (B) foreskin tissues was determined by quantitative immunofluorescent microscopy. Soluble E-cadherin was measured using ELISA (C). Student’s T test, α = 0.05. Lines shown for mean in each group. (DOCX) [file ppat.1013094.s005.docx]

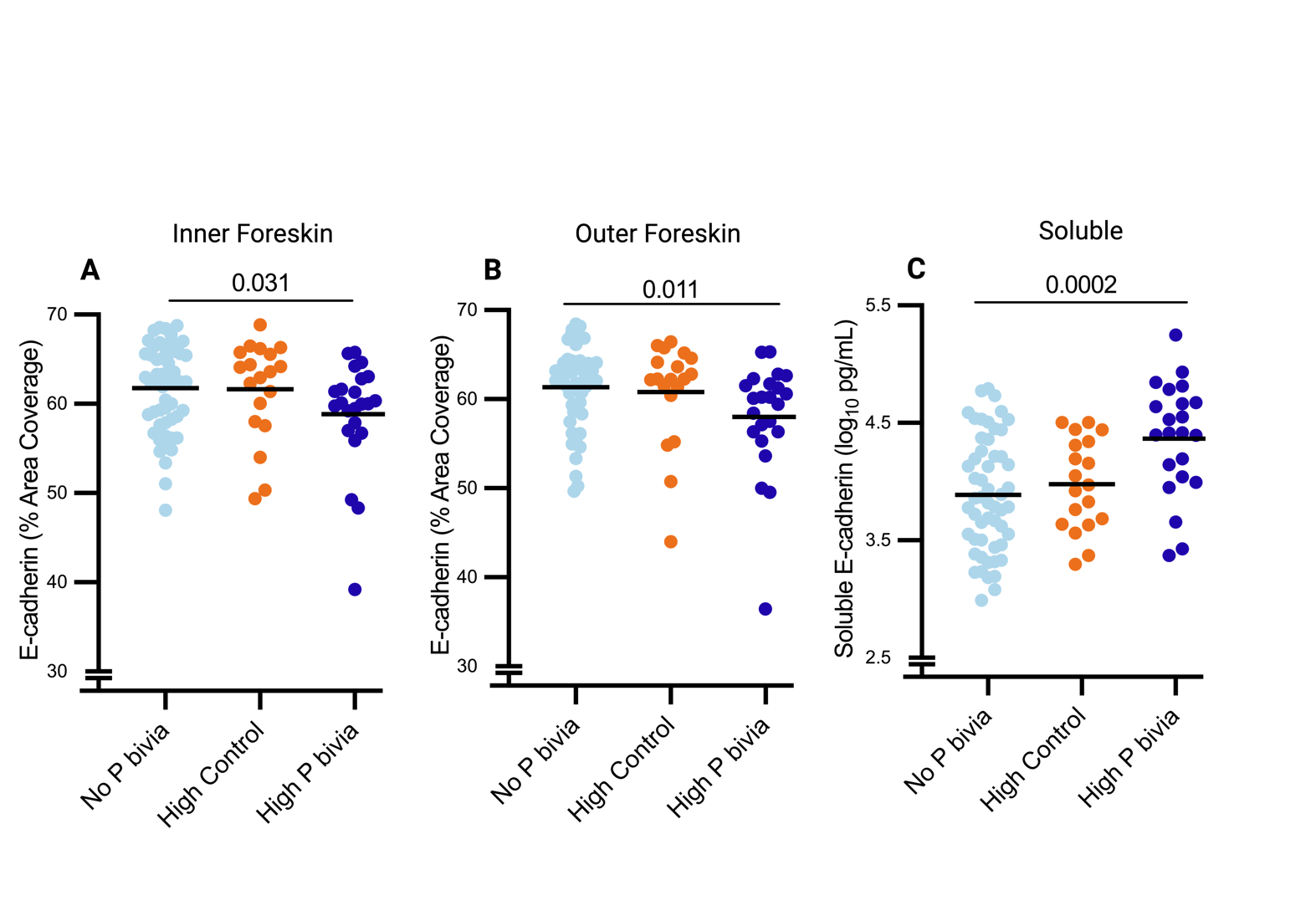


**S3 Appendix. E-cadherin Expression by *Prevotella bivia* Absolute Abundance.** Absolute abundance of *Prevotella bivia* (16S rRNA sequencing and qPCR) was determined from penile swabs. Inner and outer foreskin tissue samples were divided into 3 groups; No *Prevotella bivia* (n=54), High Control (n=19), and High *Prevotella bivia* (n=23). Relative expression of the epithelial junction protein E-cadherin in inner (**A**) and outer (**B**) foreskin tissues was determined by quantitative immunofluorescent microscopy. Soluble E-cadherin was measured using ELISA (**C**). Student’s T test, α = 0.05. Lines shown for mean in each group.
